# Clinical evaluation of analytical variations in serum creatinine measurements: why laboratories should abandon Jaffe techniques

**DOI:** 10.1186/1471-2369-13-133

**Published:** 2012-10-08

**Authors:** Iefke Drion, Christa Cobbaert, Klaas H Groenier, Cas Weykamp, Henk JG Bilo, Jack FM Wetzels, Nanne Kleefstra

**Affiliations:** 1 Diabetes Centre, Isala Clinics, P.O. Box 10400, 8000, GK Zwolle, the Netherlands; 2 Department of Clinical Chemistry and Laboratory Medicine, Leiden University Medical Center, Leiden, The Netherlands; 3 Department of General Practice, University Medical Center Groningen, Groningen, The Netherlands; 4 European Reference Laboratory, Streekziekenhuis Koningin Beatrix, Winterswijk, The Netherlands; 5 Department of Internal Medicine, University Medical Center Groningen, Groningen, The Netherlands; 6 Department of Internal Medicine, Isala Clinics, Zwolle, The Netherlands; 7 Department of Nephrology, Radboud University Nijmegen Medical Center, Nijmegen, The Netherlands; 8 Langerhans Medical Research Group, Zwolle, The Netherlands

**Keywords:** Calibration, Glomerular filtration rate, Isotope dilution-mass spectrometry, Renal function, Reference standards, Traceability

## Abstract

**Background:**

Non-equivalence in serum creatinine (SCr) measurements across Dutch laboratories and the consequences hereof on chronic kidney disease (CKD) staging were examined.

**Methods:**

National data from the Dutch annual external quality organization of 2009 were used. 144 participating laboratories examined 11 pairs of commutable, value-assigned SCr specimens in the range 52–262 μmol/L, using Jaffe or enzymatic techniques. Regression equations were created for each participating laboratory (by regressing values as measured by participating laboratories on the target values of the samples sent by the external quality organization); area under the curves were examined and used to rank laboratories. The 10^th^ and 90^th^ percentile regression equation were selected for each technique separately. To evaluate the impact of the variability in SCr measurements and its eventual clinical consequences in a real patient population, we used a cohort of 82424 patients aged 19–106 years. The SCr measurements of these 82424 patients were introduced in the 10^th^ and 90^th^ percentile regression equations. The newly calculated SCr values were used to calculate an estimated glomerular filtration rate (eGFR) using the 4-variable Isotope Dilution Mass Spectrometry traceable Modification of Diet in Renal Disease formula. Differences in CKD staging were examined, comparing the stratification outcomes for Jaffe and enzymatic SCr techniques.

**Results:**

Jaffe techniques overestimated SCr: 21%, 12%, 10% for SCr target values 52, 73 and 94 μmol/L, respectively. For enzymatic assay these values were 0%, -1%, -2%, respectively. eGFR using the MDRD formula and SCr measured by Jaffe techniques, staged patients in a lower CKD category. Downgrading to a lower CKD stage occurred in 1-42%, 2-37% and 12–78.9% of patients for the 10^th^ and 90^th^ percentile laboratories respectively in CKD categories 45–60, 60–90 and >90 ml/min/1.73 m^2^. Using enzymatic techniques, downgrading occurred only in 2-4% of patients.

**Conclusions:**

Enzymatic techniques lead to less variability in SCr measurements than Jaffe techniques, and therefore result in more accurate staging of CKD. Therefore the specific enzymatic techniques are preferably used in clinical practice in order to generate more reliable GFR estimates.

## Background

Serum creatinine (SCr) based prediction equations are frequently used in screening and clinical settings in order to estimate the glomerular filtration rate (GFR). The current variability in SCr measurements affects all estimating equations for GFR, including the MDRD equation. Many automated routine methods for SCr measurement exceed the desirable imprecision criterion of ≤ 2.2%; therefore, reduction of analytical bias ≤ 3.4% in creatinine assays by standardization of calibration is needed [1]. It is important to notice that standardization of calibration does not correct for analytical interferences (nonspecificity bias). The bias and nonspecificity problems associated with some of the routine methods must be addressed.

Chronic kidney disease (CKD) staging directly relies on these estimated GFR values. Using accurate SCr measurements is essential, since systematic errors cause unreliable renal function estimates, leading to incorrect drug dose adjustments, misclassifications in CKD staging and incomparability of patient data [2-5].

Since significant interlaboratory variation was observed worldwide, it was internationally confirmed that calibration traceability to higher-order reference methods was needed to realize comparable biochemical measurement results [2,6,7].

Therefore, the European in vitro diagnostics (IVD) directive 98/79/EC, and the laboratory working group of the National Kidney Disease Education Program recommend that in order to improve standardization, clinical laboratory measurements should be traceable to internationally recognized and certified reference materials [8-10]. Since the development of NIST SRM 967 in 2006, a matrix-based IDMS targeted creatinine standard, all essential elements (i.e. reference methods, reference laboratories, and materials) needed to complete the creatinine reference system are in place, according to ISO 17511 [11]. Because the complete traceability train is agreed upon in vitro diagnostic manufactures of creatinine assays in Europe are legally obliged to make their products metrologically traceable, regardless of the method applied.

In this study we examine the degree of variability and interchangeability of SCr measurements across all clinical chemistry laboratories in 2009 in the Netherlands, in order to evaluate the situation after global restandardization, using data from the annual national external quality control program of the Dutch external quality assessment (EQA) organization for clinical chemistry laboratories (Stichting Kwaliteitsbewaking Medische Laboratoriumdiagnostiek, SKML). Subsequently, we investigate in a theoretical model, the impact of the variability in SCr measurements between laboratories on estimates of GFR using the 4-variable IDMS-traceable MDRD formula and the consequences hereof on CKD staging of patients, when the data from the SKML are extrapolated to a large cohort of patients.

## Methods

In this cross-sectional study, we evaluate the effect of different SCr assays on SCr levels and CKD classification. EQA data are derived from the 2009 EQA program of the SKML. Annually, the SKML creates 11 pairs of frozen commutable, value assigned serum samples spiked with crystalline creatinine, aliquoted in 1 ml vials. A commutable material reflects the characteristics and properties of native patient samples [2,12]. Value-assignment was performed by a joint committee on traceability in laboratory medicine (JCTLM)-endorsed reference laboratory. Each of the 144 laboratories participating in the EQA program in the Netherlands annually receives a set containing 11 pairs of these commutable samples from the SKML and store them intermittently at −80°C. The 11 pairs of EQA-samples cover SCr values in the entire measuring range: 52-73-94-115-136-157-178-199-220-241-262 μmol/L and form a linear sequence; thus each laboratory received and analyzed, the range mentioned before in twofold over the year. The target values for the SCr levels are established by a JCTLM listed reference laboratory (Bonn, Germany) using an Isotope Dilution Gas Chromatography/Mass Spectrometry (ID-GC/MS) method [13,14].

Every other week all routine laboratories thawed one of the EQA samples and measured the SCr concentration applying their routine SCr methods according to the manufacturer’s instructions. 91 (63%) versus 48 (33%) of the laboratories used a Jaffe or enzymatic method to measure SCr, respectively. 62 (68%) laboratories using a Jaffe technique applied a modified kinetic Jaffe method; 29 (32%) used a compensated Jaffe method. Few laboratories used dry chemistry to measure SCr; since this group of laboratories was too small to draw conclusions from (n = 5), this group was excluded from further analyses. Companies and instruments included Abbott (Abott Park, Il, USA; Aeroset, Architect), Beckman (Brea, Ca, USA; Synchron, Unicel, LX20, Lxi725), Siemens Healthcare diagnostics (The Netherlands; ADVIA 1650, 1800, 2400), Roche Diagnostics (Mannheim, Germany; Integra, Hitachi, Modular, Cobas, Cobas Integra) and Olympus (Tokyo, Japan; AU 400, 600). In total, 39 different instrument method combinations were used to measure SCr.

Data of the SCr measurements as measured by the participating laboratories were reported centrally to the SKML and collected in an completely anonymous database.

### Variability SCr extrapolated in cohort

To investigate the impact of the variability in SCr measurements as found in the national EQA database and the eventual clinical consequences hereof in a real patient population, we used an unselected cohort of 82424 patients whose SCr had been measured in 2009 in the Isala Clinics Zwolle, the Netherlands; the details of this population have been described before [15]. In short, 45.3% of the population was male, age varied from 19–106 years and 38.7% was older than 65 years old. SCr in this cohort was measured using an enzymatic technique (modular P Analyzer, creatinine plus assay; Roche Diagnostics, Mannheim, Germany). In order to obtain SCr reference values that are traceable to the reference data from the EQA program for each patient, we requested the results from the 2009 EQA program from the clinical chemistry department, Isala clinics Zwolle. Based on these results we created a regression equation for the Zwolle laboratory (the exact procedure is extensively described in the statistical analyses section), using inverse regression. Subsequently SCr values as measured in the Zwolle population were introduced in this regression equation in order to calculate the SCr values traceable to the results of the EQA program for each of the 82424 patients. The GFR using these IDMS-traceable SCr values was estimated using the 4-variable IDMS-traceable MDRD formula [16,17].

### Statistical analysis

We used SPSS version 16.0 (SAS Institute, Cary, NC, USA) and STATA version 11 (StataCorp, College Station, Texas USA) for statistical analyses. All SCr measurements of the laboratories participating in the EQA program were inspected for outliers (truncated at ± 3 standard deviation (SD)); 3 laboratories were removed from the dataset because more than 50% of the measurements of these 3 laboratories deviated more than 3 SD from the other laboratories. The target reference values from the linearity sequence of the EQA program served as the reference method against which routine methods to measure SCr from participating laboratories were compared, by means of relative and absolute bias. The results were displayed in box and whisker plots for each method group. Relative bias is defined as the mean percentage difference [(measured SCr-target value SCr)/target value SCr] x 100; absolute bias is defined as the mean difference between SCr values measured by individual laboratories and SCr target values; precision is defined as the SD of the absolute bias.

We extrapolated the impact of the non-equivalence in SCr measures (as derived from the laboratories participating in the EQA program), to our patient cohort of 82424 patients. In order to do so, SCr values as measured by laboratories participating in the quality assessment program were regressed on the target values of the samples sent by the SKML, so-called inverse regression, for each of the participating laboratories separately. Regression equations for each of the participating laboratories, (n = 47 for Jaffe and n = 39 for enzymatic), who had not changed their technique to measure SCr in 2009, were created. For each of these regression equations (thus for each of the laboratories fulfilling the criteria mentioned above), we calculated an area under the curve (AUC) in the range 73–115 μmol/L. The range of 73–115 μmol/L was chosen since especially these values of SCr provide eGFR’s around the threshold value of 60 ml/min/1.73 m^2^, sufficient to classify patients as having CKD stage 3. Subsequently, the AUC’s of all the participating laboratories were ranked in ascending order for the Jaffe and the enzymatic technique separately, in order to establish a 10^th^ and 90^th^ percentile regression equation for each of the techniques. Then, SCr values from our cohort of 82424 patients were inserted in the 10^th^ and 90^th^ percentile regression equations (for the Jaffe technique and the enzymatic technique as appropriate). These ‘newly calculated’ SCr values were introduced in the appropriate MDRD equations, thus providing 10^th^ and 90^th^ percentile eGFR values. To get an impression from the clinical implications of the variation in SCr values on CKD staging, we classified the patients according to the K/DOQI guidelines and evaluated the differences in CKD staging when SCr values were measured by Jaffe or the enzymatic methods [18].

### Ethical statement

No permission was required from the Medical Ethics Committee as our data only included lab result information, which had been obtained from a laboratory database. No personal patient information was included. Permission to use the national 2009 EQA-data was obtained from the SKML. The laboratories in the dataset were anonymous.

## Results

**Figure 1:**
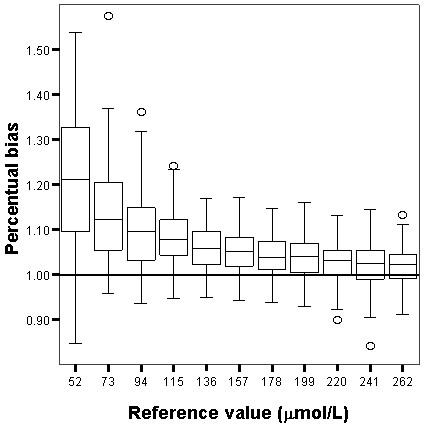
**Box and whisker plot showing the percentual bias of the Jaffe technique.** Box and whisker plot showing the percentual bias for the Jaffe technique . Interpretation of the vertical axis e.g. 1.1 means a percentual bias of 10%. The box represents the 25^th^, 50^th^ and 75^th^ percentile; the whiskers represent the 5th and 95th percentile. The extremes, defined as values more than three times the interquartile range, are the signs above and underneath the whiskers. The grey line represents the 0% bias line.

**Figure 2:**
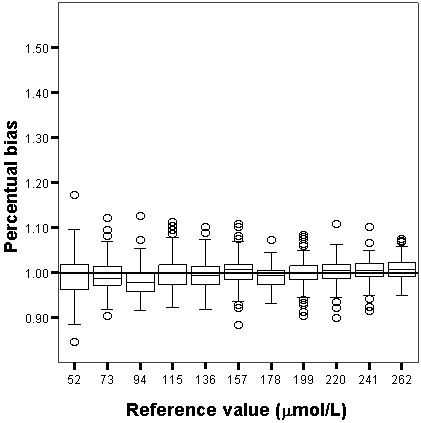
**Box and whisker plot showing the percentual bias of the enzymatic technique.** Box and whisker plot showing the percentual bias for the enzymatic technique (1). Interpretation of the vertical axis e.g. 1.1 means a percentual bias of 10%. The box represents the 25^th^, 50^th^ and 75^th^ percentile; the whiskers represent the 5th and 95th percentile. The extremes, defined as values more than three times the interquartile range, are the signs above and underneath the whiskers. The grey line represents the 0% bias line.

**Figure 3:**
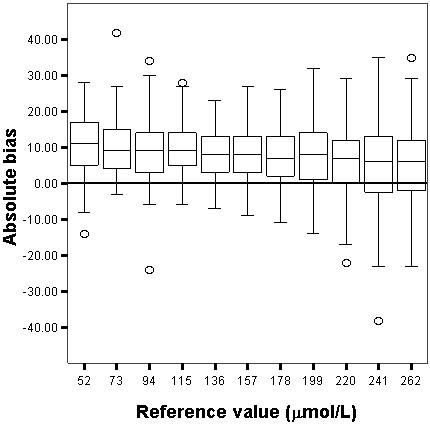
**Box and whisker plot showing the absolute bias (μmol/L) for the Jaffe technique.** The box represents the 25^th^, 50^th^ and 75^th^ percentile; the whiskers represent the 5th and 95th percentile. The extremes, defined as values more than three times the interquartile range, are the signs above and underneath the whiskers. The grey line represents the 0 μmol/L bias line.

**Figure 4:**
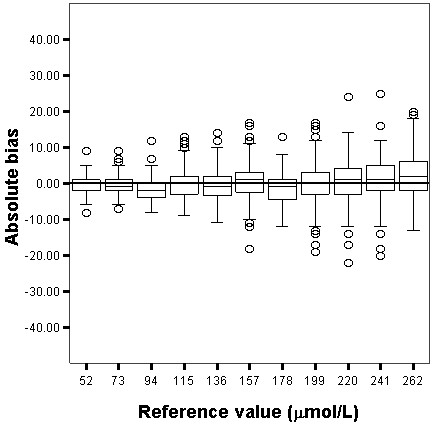
**Box and whisker plot showing the absolute bias (μmol/L) for the enzymatic technique.** The box represents the 25^th^, 50^th^ and 75^th^ percentile; the whiskers represent the 5th and 95th percentile. The extremes, defined as values more than three times the interquartile range, are the signs above and underneath the whiskers. The grey line represents the 0 μmol/L bias line.

The relative and absolute bias for both Jaffe and enzymatic techniques are shown in Figures 1, 2, 3 and 4. The enzymatic method to measure SCr produced the least biased results, which were not significantly different from the target values, whereas the Jaffe technique produced the most biased and imprecise results, which differed significantly from the reference values. The Jaffe technique especially tended to overestimate SCr at low concentrations: 21%, 12%, 10% for the SCr target values 52, 73 and 94 μmol/L, respectively. The enzymatic method had a small bias that was constant over the entire range of SCr values. The precision for Jaffe/enzymatic (per reference value) is: 10/3 (52 μmol/L), 10/3 (73 μmol/L), 7/3 (94 μmol/L), 13/4 (115 μmol/L), 7/5 (136 μmol/L), 8/4 (157 μmol/L), 8/5 (178 μmol/L), 9/5 (199 μmol/L), 10/6 (220 μmol/L), 11/5 (241 μmol/L), 5/2 (262 μmol/L) for both the Jaffe and the enzymatic method.

The impact of the variability in SCr measurements on CKD staging is illustrated in Tables 1 and 2. From the tables we can conclude that the differences between the 10^th^ and 90^th^ percentile laboratory are large when a Jaffe technique is used. Downgrading to a lower CKD class was observed using the Jaffe assay for CKD stages: 45–60 ml/min/1.73 m^2^ (1.1%, 41.9%); 60–90 ml/min/1.73 m^2^ (1.8, 36.7%) and >90 ml/min/1.73 m^2^ (12.3%, 78.9%), for the 10^th^ and 90^th^ percentile values respectively. When an enzymatic technique was used, the variability resulted in both upward and downward reclassification of CKD stage. Downward reclassification occurred in 2.1-4.1% of patients, whereas upgrading occurred in 15.6-30.1% of patients.

**Table 1:** Implications for CKD staging when a Jaffe or a standardized serumcreatinine is used

		**MDRD Jaffe p**_**10**_**/ p**_**90**_										
		**<30 ml/min/1.73 m**^**2**^		**30-45 ml/min/1.73 m**^**2**^		**45-60 ml/min/1.73 m**^**2**^		**60-90 ml/min/1.73 m**^**2**^		**>90 ml/min/1.73 m**^**2**^		**Total**
		**p**_**10**_	**p**_**90**_	**p**_**10**_	**p**_**90**_	**p**_**10**_	**p**_**90**_	**p**_**10**_	**p**_**90**_	**p**_**10**_	**p**_**90**_	
**MDRD standardized**	<30 ml/min/1.73 m^2^	2523 (99.2%)	2543 (100%)	20 (0.8%)	0 (0%)	0 (0%)	0 (0%)	0 (0%)	0 (0%)	0 (0%)	0 (0%)	2543
	30-45 ml/min/1.73 m^2^	0 (0%)	678 (17.3%)	3908 (100%)	3231 (82.7%)	1 (0%)	0 (0%)	0 (0%)	0 (0%)	0 (0%)	0 (0%)	3909
	45-60 ml/min/1.73 m^2^	0 (0%)	0 (0%)	90 (1.1%)	3439 (41.9%)	8126 (98.9%)	4777 (58.1%)	0 (0%)	0 (0%)	0 (0%)	0 (0%)	8216
	60-90 ml/min/1.73 m^2^	0 (0%)	0 (0%)	0 (0%)	0 (0%)	736 (1.8%)	14598 (36.7%)	39085 (98.2%)	25223 (63.3%)	0 (0%)	(0%)	39821
	>90 ml/min/1.73 m^2^	0 (0%)	0 (0%)	0 (0%)	0 (0%)	0 (0%)	0 (0%)	3448 (12.3%)	22047 (78.9%)	24487 (87.7%)	5888 (21.1%)	27935
		2523	3221	4018	6670	8863	19375	42533	47270	24487	5888	82424

Crosstabulation showing the estimated glomerular filtration rate (GFR) stages when the modification of diet in renal disease equation (MDRD) is calculated with a standardized serumcreatinine versus a serumcreatinine as measured by a p10 (10th percentile) or a p90 (90th percentile) laboratory using a Jaffe technique.

**Table 2:** Implication for CKD staging when an enzymatic or a standardized serumcreatinine is used

		**MDRD enzymatic p**_**10**_**/ p**_**90**_										
		**<30 ml/min/1.73 m**^**2**^		**30-45 ml/min/1.73 m**^**2**^		**45-60 ml/min/1.73 m**^**2**^		**60-90 ml/min/1.73 m**^**2**^		**>90 ml/min/1.73 m**^**2**^		**Total**
		**p**_**10**_	**p**_**90**_	**p**_**10**_	**p**_**90**_	**p**_**10**_	**p**_**90**_	**p**_**10**_	**p**_**90**_	**p**_**10**_	**p**_**90**_	
**MDRD standardized**	<30 ml/min/1.73 m^2^	2483 (97.6%)	2543 (100%)	60 (2.4%)	0 (0%)	0 (0%)	0 (0%)	0 (0%)	0 (0%)	0 (0%)	0 (0%)	2543
	30-45 ml/min/1.73 m^2^	0 (0%)	141 (3.6%)	3299 (84.4%)	3768 (96.4%)	610 (15.6%)	0 (0%)	0 (0%)	0 (0%)	0 (0%)	0 (0%)	3909
	45-60 ml/min/1.73 m^2^	0 (0%)	0 (0%)	0 (0%)	338 (4.1%)	5740 (69.9%)	7878 (95.9%)	2476 (30.1%)	0 (0%)	0 (0%)	0 (0%)	8216
	60-90 ml/min/1.73 m^2^	0 (0%)	0 (0%)	0 (0%)	0 (0%)	0 (0%)	845 (2.1%)	28308 (71.1%)	38976 (97.9%)	11513 (28.9%)	0 (%)	39821
	>90 ml/min/1.73 m^2^	5 (0%)	0 (0%)	0 (0%)	0 (0%)	0 (0%)	0 (0%)	0 (0%)	1502 (5.4%)	27930 (100%)	26433 (94.6%)	27935
		2488	2684	3359	4106	6350	8723	30784	40478	39443	26433	82424

Crosstabulation showing the estimated glomerular filtration rate (GFR) stages when the modification of diet in renal disease equation (MDRD) is calculated with a standardized serumcreatinine versus a serumcreatinine as measured by a p10 (10th percentile) or a p90 (90th percentile) laboratory using an enzymatic technique.

## Discussion

The present study shows that interlaboratory non-equivalence in SCr assays in the Netherlands was still substantial in 2009, notwithstanding the recent international creatinine restandardization effort. The high variability was largely explained by the ongoing use of Jaffe assays for measuring SCr. Compared to the enzymatic assays, the Jaffe assays had a significantly larger bias, especially for SCr levels in the lower range (reference value range 52–115 μmol/L). Although relative bias decreased when SCr reference values were higher, imprecision remained high. It was of course to be expected that Jaffe methods lead to a positive bias compared to the ID-/GC-MS method, and that adjustment for this bias would occur when using the appropriate MDRD equation. This has caused the downgrading of patients to a lower CKD category relevantly more often when a Jaffe technique instead of an enzymatic technique was used, especially in categories >45 ml/min/1.73 m^2^ (up to 79%). In contrast, the use of an enzymatic technique more often resulted in upgrading of the CKD stage, which may be explained by the differences in bias: the Jaffe technique provided higher values of SCr, whereas the enzymatic technique provided slightly lower values.

Ever since SCr is assessed in clinical practice its accuracy has been debated [1,7,19]. Although, SCr measurements are routinely performed, it is one of the most variable laboratory tests [20]. The increasing use of eGFR in clinical practice has renewed the interest on the shortcomings of the SCr methodology [1,21-24]. Since SCr is the most important variable in the renal function estimation equations, calibration of the creatinine assays is necessary to reduce bias in these formulas. This even lead to a modification of the factor used in the MDRD-equation (from 186 to 175 for IDMS traceable creatinine). However, the way this calibration was obtained has been regularly criticized in literature, due to the fact that the formulas were modified after having recalibrated the Jaffe creatinine to an IDMS traceable enzymatic method, having deleted the intercepts since these were not statistically significant.

The substantial bias and between laboratory variance as we found in our study, has been shown in various other studies in which data from proficiency testing (PT) and EQA scheme programs were evaluated [7,25,26]. A European trueness verification study of SCr also showed large interlaboratory variability before the matrix-based SRM 967 standard was available [7]. In our study we would have expected a significantly reduced interlaboratory CV due to global restandardization on SRM 967. However, despite the European IVD directive with stricter regulations, no improvements compared to earlier studies, in which a method group SD of 2.6-11 μmol/L and a median CV of 5% at a SCr concentration of 74 μmol/L, had been reached [27]. The failure to realize amelioration of interlaboratory non-equivalence is explained by the fact that standardization does not correct for analytical non-specificity problems, as is the case with the Jaffe method [28,29]. These non-specificity problems concern the measurement of many endogenous and exogenous interfering substances such as protein, glucose and ketones when SCr is measured using a Jaffe method [28-31]. Despite many attempts to improve the performance characteristics of the Jaffe reaction, non-specificity remained [7]. This leads to overestimation of the true SCr concentration. Calibration traceability cannot solve this problem nor substitute for improvement of suboptimal routine methods.

Although the enzymatic assay to measure SCr is not free of interference from various substances, it has a better specificity than the Jaffe technique [28]. This was recently confirmed in a multicentre study evaluating IDMS-traceable enzymatic creatinine assays. It showed that the majority of enzymatic methods reached the acceptable total analytical error of 8% for SCr values as low as 36 μmol/L, when adequately calibrated against IDMS, improving the traceability and standardization of creatinine [32].

Moreover, upgrading in CKD stage as we observed when enzymatic assays were used in the MDRD formula may be less relevant in routine clinical practice than the downgrading to a lower CKD stage as occurs when a Jaffe assay was used to measure SCr. E.g. a patient whose eGFR is 57 ml/min/1.73 m^2^ (CKD stage 3a) or 62 ml/min/1.73 m^2^ (CKD stage 2) when a Jaffe respectively an enzymatic assay is used to examine SCr, probably have similar risks regarding end-stage renal disease, all-cause and cardiovascular mortality. From studies comparing the prognosis associated with the two most commonly used equations to estimate GFR (the MDRD and the Chronic Kidney Disease Epidemiology collaboration equation, CKD-EPI) during a long follow-up (≥7.5 years) we know that individuals reclassified from CKD stage 3a (eGFR 45–60 ml/min/1.73 m^2^) to no CKD had lower mortality risk than those not reclassified. Moreover, these participants had an equal risk to those classified as no CKD by both formulas [33,34].

Based on the large batch of evidence in literature showing that alkaline picrate methods are inferior methods to measure creatinine, it is time for laboratories to substitute the alkaline picrate method by enzymatic methods. Moreover, if an increasing number of laboratories apply enzymatic techniques, the number of vendors of commercial enzymatic assays will increase, leading to more competition, which will ultimately reduce the costs of these assays. To bring this in a broader perspective, more accurate and precise measurements of SCr will lead to a reduction of the source of error in GFR estimates and thus errors in the staging of renal failure. Considering the number of patients that are misclassified in this study when using an alkaline picrate technique, clinical laboratories should also consider the implications for overall health costs, since patients are referred based on creatinine based estimates of GFR [35].

Although this study is a theoretical analysis, it is one of the few illustrating the consequences of variations in SCr measurements on GFR estimation and CKD staging for the individual patient. Since the majority of Dutch laboratories is included and we have a large heterogeneous cohort of patients in which we tested our model, we are able to give a good reflection of the consequences it might have for daily clinical practice. Moreover, we have studied creatinine values on 11 different levels against a strong reference method; and the samples used, were all recent instead of remote samples, which are frequently used in other studies. Selection bias may have occurred, since laboratories with too few analyses in the external quality control program were excluded for further analysis in our patient cohort. Moreover, we applied the MDRD formula in a patient cohort with an age range from 19–106 years. This may have introduced bias since, the MDRD has only been validated for patients from 19–70 years, and underestimates the GFR in patients >70 years. However, in clinical practice, laboratories automatically report eGFR’s each time a creatinine is measured, also in patients older than 70 years, and clinical decision making is often based on these estimates.

## Conclusions

In conclusion, accurate and precise measurements of SCr are required for a more reliable estimation of GFR as support for reliable clinical decision making. Enzymatic techniques measure SCr with substantially less variability than Jaffe techniques as compared to ID-MS reference values. This leads to more reliable estimation of GFR and CKD staging. To allow improvement of reliability of eGFR, specific enzymatic techniques to measure SCr are preferable over unspecific Jaffe techniques.

## Abbreviations

CKD: Chronic Kidney Disease; SCr: Serumcreatinine; GFR: Glomerular Filtration Rate; IVD: In Vitro Diagnostics; JCTLM: Joint Committee on Traceability in Laboratory Medicine; EQA: External Quality Assessment; SKML: Stichting Kwaliteitsbewaking Medische Laboratorium Diagnostiek; IDMS: Isotope Dilution Mass Spectrometry; GCMS: Gas Chromatography Mass Spectrometry; AUC: Area under the curve.

## Competing interests

Jack Wetzels received a consultancy fee from Amgen and Genzyme, received grants from Amgen, Roche, Genzyme; received travel/accommodation/meeting expenses unrelated to activities listed from Amgen, Genzyme for the ASN 2010. Henk Bilo receives a non-restricted grant for research from Novo Nordisk, is a member of the advisory committee of Merck Sharp & Dohme. The other authors have no potential conflicts of interest or disclosures relevant to the content of the manuscript to declare.

## Authors’ contributions

I.D. researched data, contributed to the discussion and wrote the manuscript; C.C. contributed to the discussion and reviewed/edited the manuscript; K.H.G. participated in the design, researched data, contributed to the discussion; C.W. contributed to the discussion and reviewed/edited the manuscript; H.J.G.B. contributed to the discussion and reviewed/edited the manuscript; J.F.M.W. participated in the design, contributed to the discussion and reviewed/edited the manuscript; N. Kleefstra participated in the design, researched data; contributed to the discussion; reviewed/edited the manuscript. All authors read and approved the final manuscript.

## Pre-publication history

The pre-publication history for this paper can be accessed here:

http://www.biomedcentral.com/1471-2369/13/133/prepub

## References

[B1] Myers GL Miller WG Coresh J Fleming J Greenberg N Greene T Hostetter T Levey AS Panteghini M Welch M Eckfeldt JH National Kidney Disease Education Program Laboratory Working Group Recommendations for improving serum creatinine measurement: a report from the Laboratory Working group of the National Kidney Disease Education Program Clin Chem 2006 52 5 18 10.1373/clinchem.2005.0525144 16332993

[B2] Panteghini M Forest JC Standardization in laboratory medicine: new challenges Clin Chem Acta 2005 355 1 12 10.1016/j.cccn.2004.12.003 15820472

[B3] Coresh J Eknoyan G Levey AS Estimating the prevalence of low glomerular filtration rate requires attention to the creatinine assay calibration J Am Soc Nephrol 2002 13 2811 2812 10.1097/01.ASN.0000037420.89149.C9 12397055

[B4] Coresh J Astor BC McQuillan G Kusek J Greene T Van Lente F Levey AS Calibration and random variation of the serum creatinine assay as critical elements of using equations to estimate glomerular filtration rate Am J Kidney Dis 2002 39 920 929 10.1053/ajkd.2002.32765 11979335

[B5] Panteghini M Myers G Miller WG Greenberg N The importance of metrological traceability on the validity of creatinine measurement as an index of renal function Clin Chem Lab Med 2006 44 1287 1292 17032144 10.1515/CCLM.2006.234

[B6] Panteghini M Traceability, reference systems and result comparability Clin Biochem Rev 2007 28 97 104 17909614 PMC1994107

[B7] Delanghe J Cobbaert C Galteau MM Harmoinen R Jansen R Kruse R Laitinen P Thienpont LM Wuyts B Weykamp C Panteghini M Trueness verification of the current creatinine assays demonstrates a disappointing variability which insufficiently meets changing clinical needs Clin Chem Lab Med 2007 45 S59 10.1515/CCLM.2008.256 18605952

[B8] Dati F The new European directive on in vitro diagnostics Clin Chem Lab Med 2003 41 1289 1298 14580154 10.1515/CCLM.2003.196

[B9] Lex EU Directive 98/79 on in vitro medical devices Off J L 1998 331 1 37

[B10] Müller MM Implementation of reference systems in laboratory medicine Clin Chem 2000 46 1907 1909 11106321

[B11] Dodder NG Tai SS Sniegoski LT Zhang NF Welch MJ Certification of creatinine in a human serum reference material by GC-MS and LC-MS Clin Chem 2007 53 1694 1699 10.1373/clinchem.2007.090027 17660272

[B12] Franzini C Ceriotti F Impact of reference materials on accuracy in clinical chemistry Clin Biochem 1998 31 449 457 10.1016/S0009-9120(98)00054-X 9740966

[B13] Stöckl D Reinauer H Candidate reference methods for determining target values for cholesterol, creatinine, uric acid, and glucose in external quality assessment and internal accuracy control. I. Method setup Clin Chem 1993 39 993 1000 8504568

[B14] Thienpont LM Leenheer AP Stöckl D Reinauer H Candidate reference methods for determining target values for cholesterol, creatinine, uric acid, and glucose in external quality assessment and internal accuracy control. II. Method transfer Clin Chem 1993 39 1001 1006 8504529

[B15] Drion I Joosten H van Hateren KJJ Kleefstra N Krabbe JG Wetzels JF Bilo HJ Employing age-related cut-off values results in fewer patients with renal impairment in secondary care Ned Tijdschr Geneeskd 2011 155 A3091 21771358

[B16] Levey AS Bosch JP Lewis JB Greene T Rogers N Roth D A more accurate method to estimate glomerular filtration rate from serum creatinine: a new prediction equation. Modification of Diet in Renal disease study group Ann Intern Med 1999 130 461 470 10075613 10.7326/0003-4819-130-6-199903160-00002

[B17] Levey AS Greene T Kusek J Beck G A simplified equation to predict glomerular filtration rate from serum creatinine [abstract] J Am Soc Nephrol 2000 11 155a

[B18] National Kidney Foundation K/DOQI clinical practice guidelines for chronic kidney disease: evaluation, classification, and stratification Am J Kidney Dis 2002 39 S1-D246 11904577

[B19] Lawson N Lang T Broughton A Prinsloo P Turner C Marenah C Creatinine assays: time for action? Ann Clin Biochem 2002 39 599 602 10.1258/000456302760413397 12564843

[B20] Joffe M Hsu C Feldman HI Weir M Landis JR Hamm L Chronic Renal Insufficiency Cohort (CRIC) Study Group Variability of creatinine measurements in clinical laboratories: results from the CRIC study Am J Nephrol 2010 31 426 434 10.1159/000296250 20389058 PMC2883847

[B21] Stevens LA Coresh J Greene T Levey AS Assessing kidney function–measured and estimated glomerular filtration rate N Engl J Med 2006 354 2473 2483 10.1056/NEJMra054415 16760447

[B22] Levey AS Coresh J Greene T Stevens LA Zhang YL Hendriksen S Kusek JW Van Lente F Chronic Kidney Disease Epidemiology Collaboration Using standardized serum creatinine values in the modification of diet in renal disease study equation for estimating glomerular filtration rate Ann Intern Med 2006 145 247 254 16908915 10.7326/0003-4819-145-4-200608150-00004

[B23] Levey AS Coresh J Greene T Marsh J Stevens LA Kusek JW Van Lente F Chronic Kidney Disease Epidemiology Collaboration Expressing the Modification of Diet in Renal Disease Study equation for estimating glomerular filtration rate with standardized serum creatinine values Clin Chem 2007 53 766 772 10.1373/clinchem.2006.077180 17332152

[B24] Stevens LA Manzi J Levey AS Chen J Deysher AE Greene T Poggio ED Schmid CH Steffes MW Zhang YL Van Lente F Coresh J Impact of creatinine calibration on performance of GFR estimating equations in a pooled individual patient database Am J Kidney Disease 2007 50 21 35 10.1053/j.ajkd.2007.04.004 17591522

[B25] Stöckl D Libeer JC Reinauer H Thienpont LM De Leenheer AP Accuracy-based assessment of proficiency testing results with serum from single donations: possibilities and limitations Clin Chem 1996 42 469 470 8598118

[B26] Carobene A Ferrero C Ceriotti F Modenese A Besozzi M de Giorgi E Franzin M Franzini C Kienle MG Magni F Creatinine measurement proficiency testing: assignment of matrix-adjusted ID GC-MS target values Clin Chem 1997 43 1342 1347 9267311

[B27] European commission – Joint Research Centre Institute for Reference Materials and Measurements (IRMM) 2003 Geel, Belgium: Institute for Reference Materials and Measurements

[B28] Cobbaert CM Baadenhuijsen H Weykamp CW Prime time for enzymatic creatinine methods in pediatrics Clin Chem 2009 55 549 558 10.1373/clinchem.2008.116863 19168555

[B29] Panteghini M IFCC Scientific Division Enzymatic assays for creatinine: time for action Clin Chem Lab Med 2008 46 567 572 18298343 10.1515/CCLM.2008.113

[B30] Cook JG Association of clinical Biochemists’ Scientific and Technica Committee Factors influencing the assay of creatinine Ann Clin Biochem 1975 12 219 232 15637880 10.1177/000456327501200162

[B31] Cruickshank AM Ballantyne FC Shenkin A Negative interference in a kinetic Jaffe method for serumcreatinine determination Ann Clin Biochem 1988 25 112 113 3355083 10.1177/000456328802500117

[B32] Piéroni L Delanaye P Boutten A Bargnoux AS Rozet E Delatour V Carlier MC Hanser AM Cavalier E Froissart M Cristol JP Société Francaise de Biologie Clinique A multicentric evaluation of IDMS-traceable creatinine enzymatic assays Clin Chim Acta 2011 412 2070 2075 10.1016/j.cca.2011.07.012 21803031

[B33] White SL Polkinghorne KR Atkins RC Chadban SJ Comparison of the prevalence and mortality risk of CKD in Australia using the CKD epidemiology collaboration (CKD-EPI) and modification of diet in renal disease (MDRD) Study GFR estimating equations: the AusDiab (Australian Diabetes, Obesity and Lifestyle) study Am J Kidney Dis 2010 55 660 670 10.1053/j.ajkd.2009.12.011 20138414

[B34] Matsushita K Selvin E Bash LD Astor BC Coresh J Risk implications of the new CKD epidemiology collaboration (CKD-EPI) equation compared with the MDRD Study equation for estimated GFR: the Atherosclerosis risk in communities (ARIC) study Am J Kidney Dis 2010 55 648 659 10.1053/j.ajkd.2009.12.016 20189275 PMC2858455

[B35] Panteghini M The future of laboratory medicine: understanding the new pressures Clin Biochem Rev 2004 25 207 215 18458714 PMC1934959

